# Long-term high-protein diet intake reverts weight gain and attenuates metabolic dysfunction on high-sucrose-fed adult rats

**DOI:** 10.1186/s12986-018-0290-y

**Published:** 2018-07-24

**Authors:** Rosângela Maria Lopes Sousa, Nathalee Liberal Xavier Ribeiro, Bruno Araújo Serra Pinto, Jonas Rodrigues Sanches, Mariana Uchôa da Silva, Caio Fernando Ferreira Coêlho, Lucas Martins França, José Albuquerque de Figueiredo Neto, Antonio Marcus de Andrade Paes

**Affiliations:** 1 Laboratory of Experimental Physiology, Department of Physiological Sciences – DCF, Health and Biological Sciences Centre, Federal University of Maranhão – UFMA, Avenida dos Portugueses, 1966. Cidade Universitária D. Delgado, São Luís, MA 65080-805 Brazil; 2 Health Sciences Graduate Program, Health and Biological Sciences Centre, Federal University of Maranhão, São Luís, MA Brazil; 3 Department of Medicine I, Health and Biological Sciences Centre, Federal University of Maranhão, São Luís, MA Brazil

**Keywords:** High-protein diet, High-sucrose diet, Withdrawal of sucrose, Metabolic syndrome

## Abstract

**Background:**

Consumption of added sugars has been considered a worldwide public health concern by its association with metabolic syndrome and its comorbidities. Meanwhile, current studies have suggested high-protein diets to promote weight loss and improved metabolic outcomes. Thus, this study aimed to investigate the effects of long-term high-protein diet (HPD, 34.3% protein) intake on high-sucrose-fed rats.

**Methods:**

Weaned male Wistar rats were randomized into two groups: rats fed a standard chow (CT/CT, 10% sucrose) or rats fed a high-sucrose diet (HSD, 25% sucrose) for a 20-week observational period. Subsequently, HS/HS animals were randomized into 3 new groups: rats maintained on HSD diet (HS/HS); rats submitted to HSD replacement by standard chow (HS/CT); and those with HSD replaced by HPD (HS/HP). All groups were followed up for 12 weeks during which we investigated the effects of HPD on body weight, energy intake, obesity development, glicemic/lipid profile, glucose tolerance, insulin resistance, tissue weight (adipose tissue, liver and skeletal muscles), lipolytic activity, liver lipoperoxidation and histology, as well as serum markers of hepatic function.

**Results:**

Post-weaning exposure to HSD led to metabolic syndrome phenotype at adulthood, herein characterized by central obesity, glucose intolerance, dyslipidaemia and insulin resistance. Only HPD feeding was able to revert weight gain and adipose tissue accumulation, as well as restore adipose tissue lipolytic response to sympathetic stimulus. On the other hand, either HPD or withdrawal from HSD promoted very similar metabolic outcomes upon 12-week nutritional intervention. HS/HP and HS/CT rats showed reduced fasting serum levels of glucose, triacylglycerol and total cholesterol, which were correlated with the improvement of peripheral insulin sensitivity, as inferred from *k*ITT and TyG Index values. Both nutritional interventions restored liver morphofunctional patterns, but only HPD restored lipid peroxidation.

**Conclusions:**

Our data showed that 12-week intake of an isocaloric moderately high-protein diet consistently restored high-sucrose-induced central adiposity and obesity in addition to the attenuation of other important metabolic outcomes, such as improvement of glucolipid homeostasis associated to increased insulin sensitivity and reversal of hepatic steatosis. On the other hand, simple withdrawal from high-sucrose consumption also promoted the abovementioned metabolic outcomes with no impact on body weight.

**Electronic supplementary material:**

The online version of this article (10.1186/s12986-018-0290-y) contains supplementary material, which is available to authorized users.

## Background

Consumption of added sugars has been considered a worldwide public health concern by its implied association with the epidemic of obesity, type 2 diabetes mellitus (T2DM) and metabolic syndrome (MetS) [1]. Although this is still an issue of intense debate [2, 3], evidence from basic [4] and clinical [5] studies consistently support such concern. Additionally, it has been demonstrated the ability of sugar-based western diet to genetically modify the activity of thrifty genes on Pacific people, which were not historically prone to obesity [6]. This scenario becomes still worse in face of the fact that sucrose is one of the few components of our diet for which no upper limits are suggested in the majority of dietary guidelines around the globe [1], although it has been recommended a total sugar intake of 10% at maximum [7].

Sucrose intake dually promotes glucose-elicited insulin release toward its own uptake by insulin-sensitive tissues, as well as insulin-independent fructose uptake by hepatocytes and adipocytes [8]. Inside the latter, fructose is converted into fatty acids through de novo lipogenesis (DNL) that favours triacylglycerol (TAG) synthesis, very-low-density lipoprotein (VLDL) secretion and adipocyte hypertrophy [9, 10]. Sustained DNL activation in both territories intensifies lipid accumulation which also impairs insulin signalling within a vicious cycle towards non-alcoholic fatty liver disease (NAFLD) onset [11] and adipose tissue dysfunction [10]. Experimentally, we have shown that 60-day exposure of weaning rats to a high-sucrose but isocaloric diet (HSD) augmented body mass in association with increased fat accumulation in both subcutaneous and visceral depots. These animals also presented hyperglycaemia, hypertriglyceridemia, glucose intolerance and impaired insulin sensitivity [12].

Dietary manipulation is a primary factor in controlling and preventing obesity and its comorbidities. Much of the approaches have prioritized low-fat and/or low-carbohydrate interventions [13]. On the other hand, current studies have suggested high-protein diets to promote greater weight-loss and metabolic outcomes in both human beings [14, 15] and rodents [16, 17]. The main rationale behind such advantage is that dietary proteins induce the release of anorexigenic and orexigenic hormones, which ultimately modulate neuronal pathways involved in the regulation of appetite and satiety, as well as energy expenditure [17–19]. Contrariwise, some studies have shown high-protein intake effects are only temporary and subjects (human beings and rodents) regain body weight upon mid- to long-term interventions, suggesting a metabolic adaptation to high-protein regimens [20–22]. Such controversial outcomes put high-protein strategy into checkmate, deserving additional studies to support the development of differential dietary interventions to prevent or treat patients with obesity associated or not to MetS.

Therefore, in the present study we took advantage of our previously described model of MetS induced by HSD in rats [12] to assess the hypothesis that a 12-week high-protein nutritional intervention is more effective than mere excess sucrose withdrawal on the management of body weight and MetS-associated comorbidities. Our data provide a body of evidence that only long-term high protein diet (HPD) intake promotes sustained body weight management, as well as reestablishment of white adipose tissue (WAT) function, although excess sucrose withdrawal had equally improved glycemic control and dyslipidemia on those animals.

## Methods

### Experimental design

Weaned male Wistar-Hannover rats (postnatal day 21) provided by the animal facility house of the Federal University of Maranhão were randomized into two groups: control rats (CT/CT; *n* = 7), fed a standard chow (Nuvital®, Nuvilab, Brazil) composed by 55.4% total carbohydrate (10% sucrose), 21% protein, 5.2% total lipids, totalling 3.52 kcal/g; or high-sucrose diet rats (HS/HS, *n* = 18), fed a high-sucrose chow composed by 65% total carbohydrate (25% sucrose), 12.3% protein, 4.3% total lipids, totalling 3.48 kcal/g, as previously described [12], for a 20-week period. Afterwards, HS/HS animals were randomized into 3 new groups: rats maintained on HSD (HS/HS; *n* = 6); rats submitted to HSD replacement by standard chow (HS/CT; *n* = 6); and those with HSD replaced by HPD (HS/HP; *n* = 6). HPD was composed by 49% total carbohydrate (no sucrose), 34.3% protein, 2.2% total lipids, totalling 3.53 kcal/g. Micronutrient composition of each diet is described in Additional file 1: Table S1. All groups were followed up for an additional 12-week nutritional intervention, totalling a 32-week follow-up, during which they were maintained under controlled conditions (21 ± 2 °C; 60% humidity and 12 h light/dark cycle) with water and chow ad libitum.

Throughout observational period, body weight and energy intake were assessed twice a week. In parallel, Lee index ((body weight (g)^1/3^ ÷ naso-anal length (cm)) × 1000) was monthly calculated for assessment of obesity development [23]. During the 20th week of observational period, 8-h fasted rats had their tail-tip cut out and venous blood collected for fasting glucose measurement through glucometer and subsequent operation of the intraperitoneal glucose tolerance test. Collected blood samples were further processed for serum separation and measurement of total cholesterol (TC) and triglycerides (TAG) levels prior to 12-week nutritional intervention.

During the last week of nutritional intervention, animals were submitted to intraperitoneal glucose and insulin tolerance tests. By the completion of the 32-week follow-up, 8-h fasted animals were anesthetized (40:10 mg/kg ketamine:xylazine solution) for blood and tissue collection upon laparotomy. White (retroperitoneal, periepididymal and mesenteric) and brown (interscapular) adipose tissue fat pads, liver and posterior skeletal muscles (gastrocnemius and soleus) were weighed for morphometric assessment and expressed as tissue mass (g) per 100 g body weight. Periepididymal adipose tissue samples were further used for ex vivo lipolysis assay. Liver lobes were processed for histological analysis and serum samples used for determination of biochemical and hormonal profile.

All procedures were performed in accordance with the rules of Brazilian Council for the Control of Animal Experimentation (CONCEA) and approved by the Ethical Committee on Animal Use and Welfare (CEUA) of CEUMA University under the protocol number 177/17.

### Assessment of glucose-insulin axis function

For intraperitoneal glucose tolerance test (*ip*GTT), animals were submitted to 8-h fasting prior to administration of glucose 2 g/kg. Tail vein blood drops were collected immediately before (time 0) and 15, 30, 60 and 120 min after glucose load for glycemia measurement through glucometer (Accu check Active®, Roche Diagnostic, Germany). Similar procedure was carried out for intraperitoneal insulin tolerance test (*ip*ITT), excepting animals were fed and received 1 UI/kg insulin (Humulin 70/30®, Lilly, USA). Glucose disappearance rate (kITT) was derived from ITT curve and calculated as 0.693/ t_1/2_ [24]. The insulin resistance was inferred from TyG Index calculation [ln (fasting glucose (mg/dL) × fasting triglyceride (mg/dL)) / 2] [25].

### Assessment of serum biochemical profile

Serum samples were obtained upon spontaneous coagulation and centrifugation (3500 rpm; 10 min; 4 °C) and used for colorimetric measurement of TAG, TC, aminotransferases enzymes (AST and ALT), γ-glutamyl transferase, alkaline phosphatase, albumin, total protein and urea (Labtest, Brazil) levels according to manufacturer’s instructions. Insulin levels were assessed by immunoenzymatic method according to manufacturer’s instructions (Sigma-Aldrich, USA).

### Ex vivo lipolysis assay

To assess the lipolytic activity on WAT, periepididymal samples (~ 100 mg) were fragmented and incubated with Krebs buffer (120 mM NaCl, 15 mM NaHCO_3_, 4.8 mM KCl, 1.2 mM MgSO_4_, 1.2 mM KH_2_PO_4_, 2.4 mM CaCl_2_, 1% BSA and 0.1% glucose after pH adjustment to 7.4) for 1 h at 37 °C in the absence or presence of 20 μM isoproterenol (Sigma-Aldrich, USA). At the end of incubation, samples were cooled out to stop the reaction. Glycerol release (μg of glycerol/mg of tissue/h) was determined by colorimetric assay using a triglyceride assay kit according to manufacturer’s instructions (Labtest, Brazil) [26].

### Assessment of hepatic oxidative damage

To assess hepatic oxidative damage, we measured tissue levels of malondialdehyde (MDA), as previously described [27, 28]. Briefly, liver samples were homogenized in 20 mM phosphate buffer solution containing 140 mM KCl (pH 7.4; 1:10 *w*/*v*) and centrifuged (750 ×g; 10 min; 4 °C) to separate the supernatant. To avoid MDA formation during sample processing, 0.01 vol% butylated hydroxytoluene (BHT, Sigma-Aldrich, USA) and 1 mM EDTA (Sigma-Aldrich, USA) were added to the buffer. Then, 150 μL of supernatant was added to 300 μL of cold 10% trichloroacetic acid (Sigma-Aldrich, USA) and 0.67% thiobarbituric acid (Merck, Germany) in 7.1% sodium sulfate and incubated in a boiling water bath for 25 min. The mixture was cooled and the pink chromogen was isolated by addiction of buthanol (2:1 *v*/v) followed by centrifugation (3000 ×g; 10 min; 4 °C). The resulting pink chromogen TBARS were determined spectrophotometrically at 535 nm. Calibration curve was performed using 1,1,3,3-tetramethoxypropane (Sigma-Aldrich, USA) as standard. TBARS levels were expressed as μM of MDA/mg protein.

### Histological analysis

Samples from hepatic tissue were fixed in 10% phosphate-buffered formalin solution and they were analysed by light microscopy either after hematoxilin-eosin (HE) or Masson’s Trichrome staining. NAFLD activity score (NAS) [29] was applied for semi-quantitative analysis of the three definer criteria of non-alcoholic steatohepatitis: steatosis (0–3), ballooning (0–3), and lobular inflammation (0–2).

### Statistical analysis

The sample size was calculated by using the free software G* Power 3.1 (Heinrich-Heine University Düsseldorf, Düsseldorf (NRW), Germany) [30] and on the basis of the effects of HSD on body weight gain, as well as plasma parameters from previous studies [12, 31, 32]. In accordance, a sample size of six animals per group was found to provide the appropriate power (1 − β = 0·8) to identify significant differences (α = 0·05) in the variables analysed. Statistical analysis was conducted using GraphPad Prism 7.0 software (GraphPad Software Inc., USA). Data were expressed as mean ± SEM and submitted to normality test (Kolmogorov-Smirnov) followed by parametrical analysis through unpaired *t* test (one-tailed) or one-way ANOVA (post-test Newman-Keuls) for a significance level of 5% (*p* < 0.05).

## Results

### Post-weaning exposure to high-sucrose diet leads to metabolic syndrome phenotype at adulthood

**Fig. 1:**
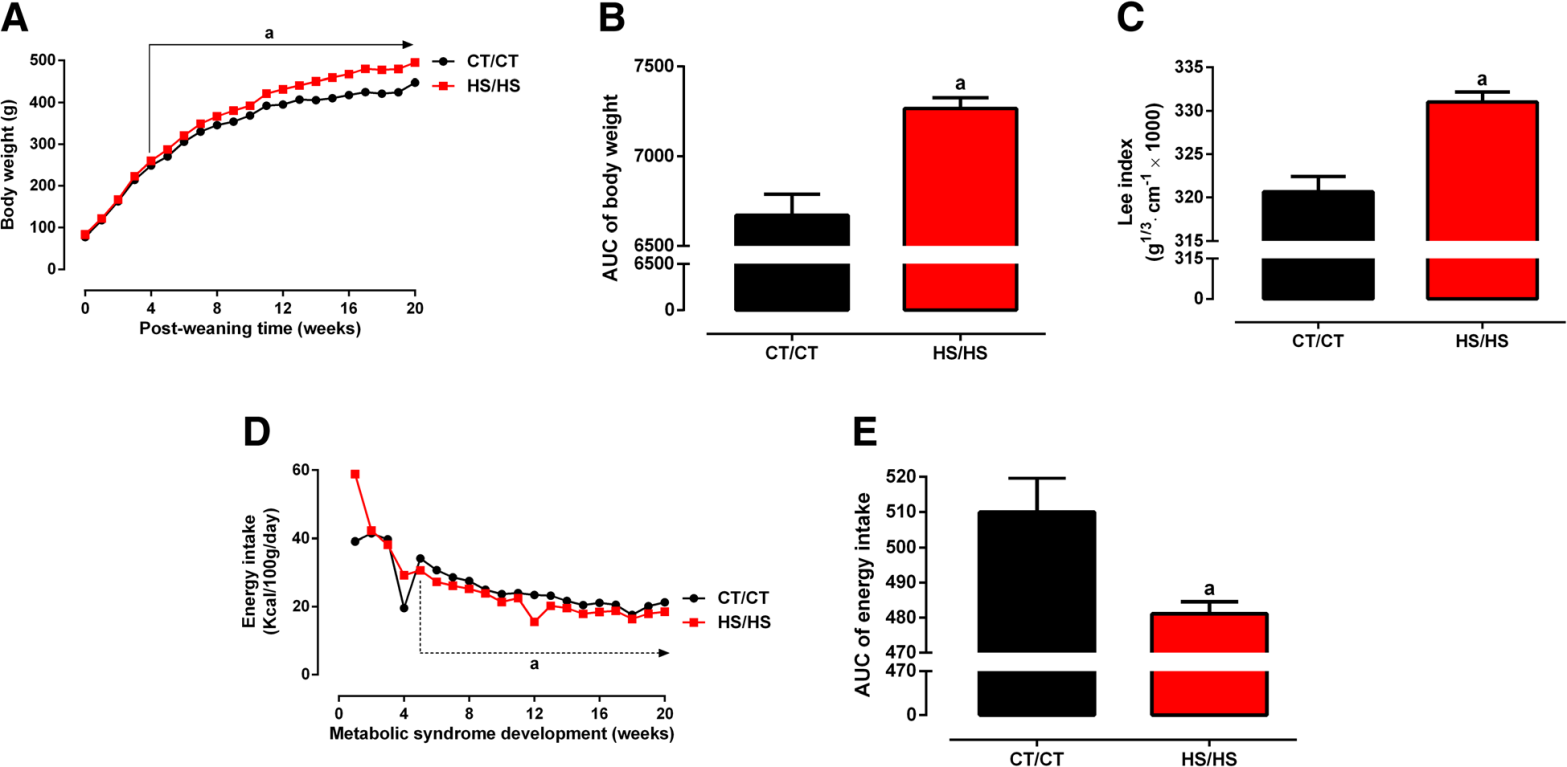
Morphometric parameters before nutritional intervention. **a** Body weight (g); **b** area under curve (AUC) of body weight; **c** Lee index (g^1/3^ cm^− 1^ × 1000); **d** energy intake (Kcal/100 g/day); and **e** AUC of energy intake were assessed in rats fed a standard chow (CT/CT, *n* = 7) and high-sucrose diet (HS/HS, *n* = 18) during 20 weeks from weaning (postnatal day 21). Points and bars represent mean ± SEM, compared by unpaired t-test (one-tailed). ^a^ represents *p* < 0.05 when compared to CT/CT

Male rats exposed to HSD from weaning up to adulthood (24-weeks-old) evolved over time to a metabolic syndrome phenotype. As showed in Fig. 1a, HS/HS rats presented higher body weight than CT/CT from 4th post-weaning week (260.0 ± 2.8 g vs. 248.6 ± 4.2 g, *p* = 0.038), reaching a 10% higher body weight at 20th post-weaning week (495.0 ± 4.1 g vs. 447.8 ± 9.4 g, *p* < 0.0001). A proportional increase was observed when analysed the area under the body weight curve (Fig. 1b). Assessment of body mass through Lee Index calculation showed HS/HS rats were obese, as compared to CT/CT (331.0 ± 1.2 vs. 320.7 ± 1.7 g^1/3^ × cm^− 1^, *p* < 0.0001, Fig. 1c). Interestingly, HS/HS rats developed an obese phenotype in spite of having a decreased energy intake, which was statistically lower CT/CT rats from the 5th post-weaning week (Fig. 1d, e).

**Fig. 2:**
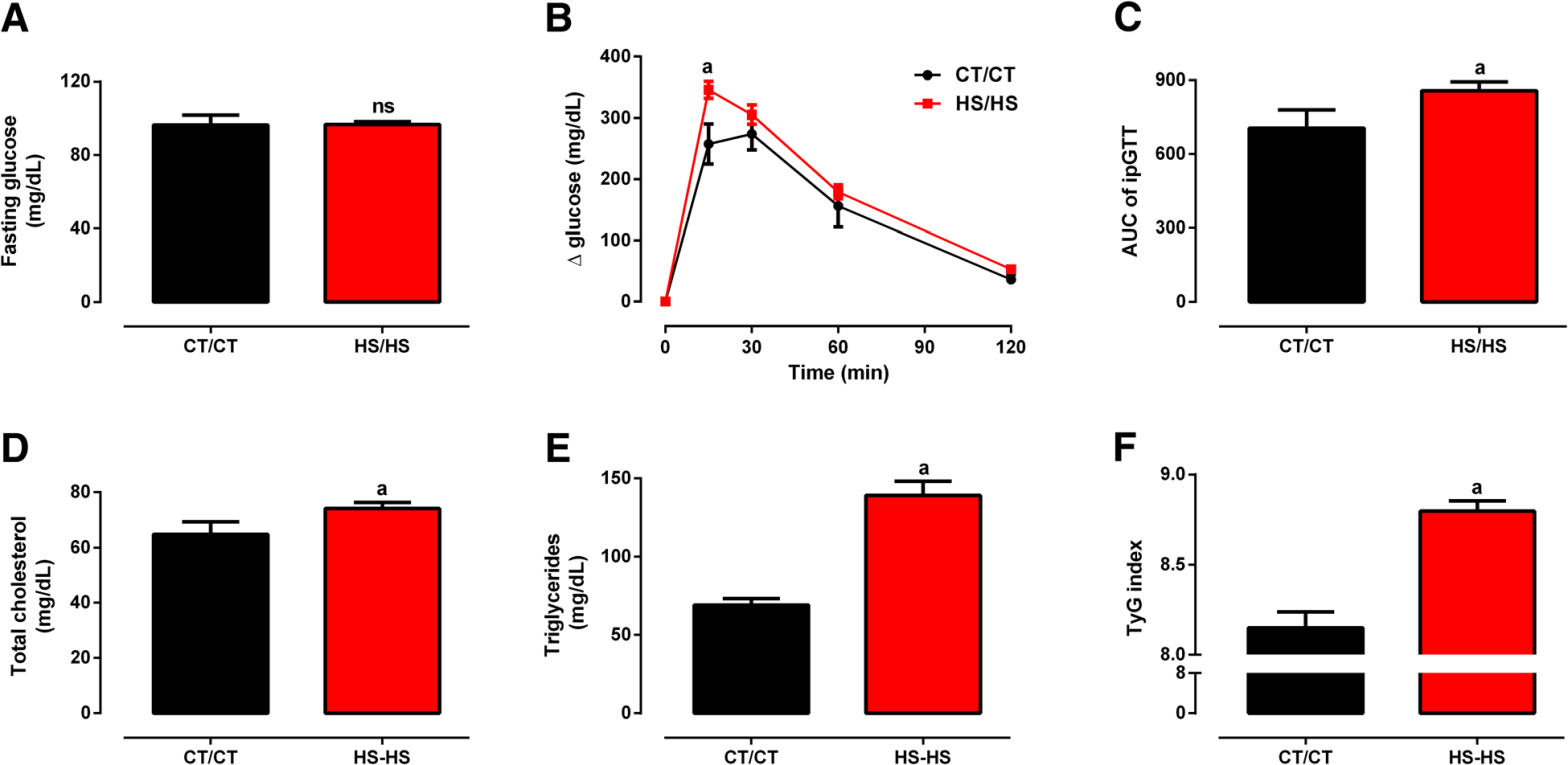
Glycemic/lipid profile and insulin resistance before nutritional intervention. **a**, blood glucose levels (mg/dL) in fasting state; **b**, blood glucose levels (mg/dL) during glucose tolerance test (GTT); **c**, AUC of GTT; **d**, serum total cholesterol levels (mg/dL); **e**, serum triglycerides levels (mg/dL); **f**, TyG index assessed in rats fed a standard chow (CT/CT, *n* = 7) and high-sucrose diet (HS/HS, *n* = 18) during 20 weeks from weaning (postnatal day 21). Points and bars represent mean ± SEM, compared by unpaired t-test (one-tailed). ^ns^ represents *p* > 0.05 and ^a^ represents *p* < 0.05 when compared to CT/CT

Long-term HSD consumption negatively impacted on glucose and lipid homeostasis. HS/HS rats did not present fasting hyperglycaemia (Fig. 2a), but had impaired glucose tolerance in comparison to CT/CT (Figs. 2b, c), *p* < 0.05. Serum TC levels were slightly augmented in HS/HS (74.1 ± 2.2 mg/dL) when compared to CT/CT (64.7 ± 4.6 mg/dL, Fig. 2d), *p* < 0.05; whereas serum TAG levels were 2-fold higher in HS/HS than CT/CT rats (139.1 ± 9.1 vs. 69.1 ± 4.1 mg/dL, Fig. 2e), *p* < 0.0001. HS/HS rats also presented insulin resistance, since TyG Index values for this group (8.8 ± 0.05) were significantly higher than those obtained for CT/CT (8.1 ± 0.09, Fig. 2f), *p* < 0.0001. Overall, this set of data consistently demonstrate that early introduction of dietary sugars leads to metabolic syndrome at adulthood, herein characterized by presence of the following metabolic outcomes: obesity, glucose intolerance, dyslipidaemia and insulin resistance.

### High-protein diet reverts the weight gain induced by high-sucrose diet consumption

**Fig. 3:**
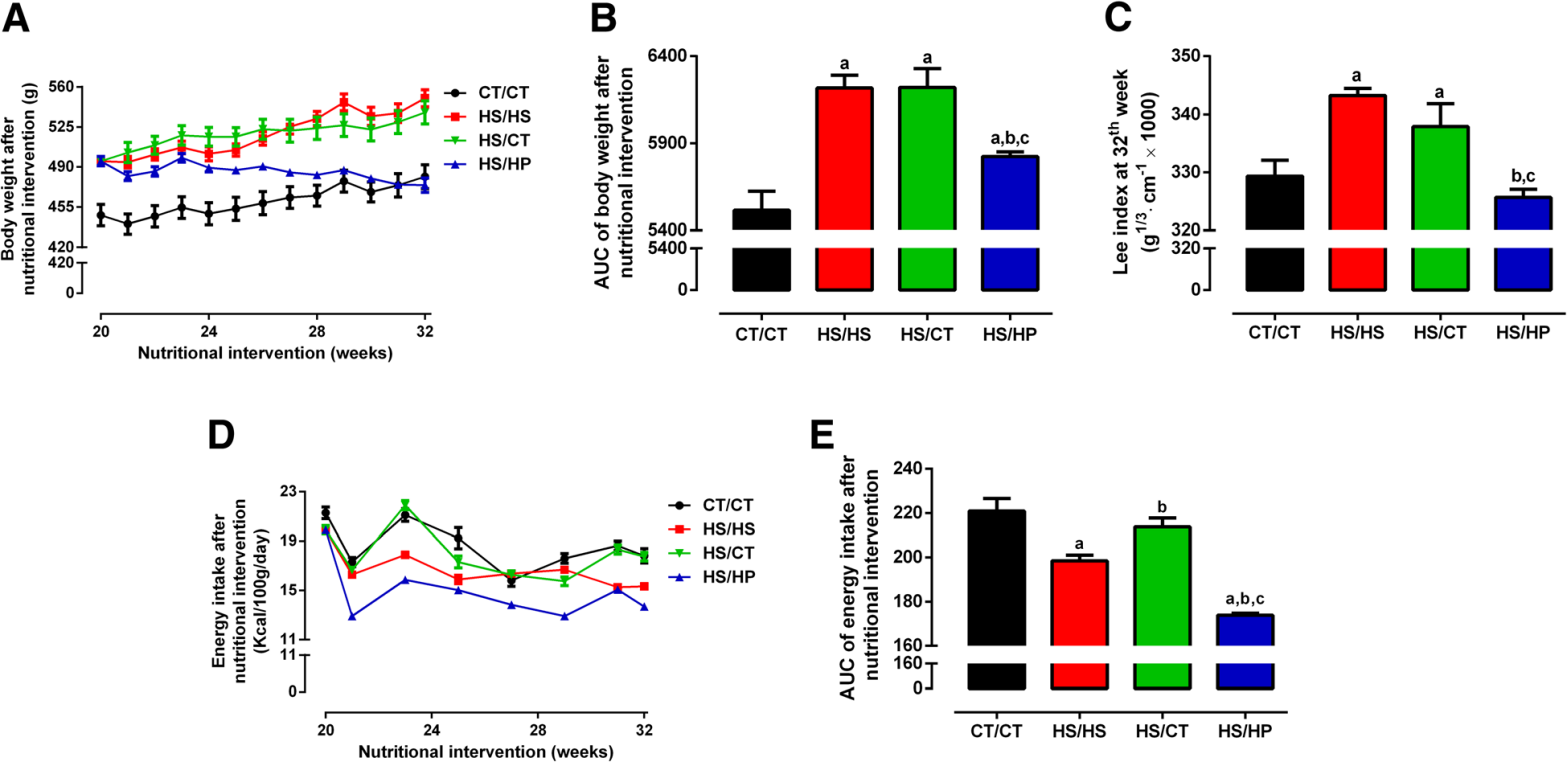
Morphometric parameters after nutritional intervention. **a**, Body weight (g); **b**, area under curve (AUC) of body weight; **c**, Lee index (g^1/3^ cm^− 1^ × 1000); **d**, energy intake (Kcal/100 g/day) and **e**, area under curve (AUC) of energy intake assessed in rats fed a standard chow (CT/CT, *n* = 7), high-sucrose diet (HS/HS, *n* = 6), initially fed a HS diet and then replaced by standard chow (HS/CT, *n* = 6) and fed a HS diet then replaced by high-protein diet (HS/HP, *n* = 6). The nutritional intervention started after 20 weeks post weaning and lasted for 12 weeks. Points and bars represent mean ± SEM, compared by one-way ANOVA (Newman Keuls). ^a^ represents *p* < 0.05 when compared to CT/CT, ^b^ represents *p* < 0.05 when compared to HS/HS and ^c^ represents *p* < 0.05 when compared to HS/CT

We next assessed the effects of a 12-week nutritional intervention with HPD on HSD-fed rats. HS/HP rats lose weight from 495.0 ± 4.1 g to 475.0 ± 6.3 g (*p* < 0.0001), no longer differing from CT/CT (481.5 ± 10.4 g; Fig. 3a). On the other hand, HS/HS and HS/CT rats maintained similar upward trend of weight gain (Fig. 3a). The effect of HPD on weight loss is better seen when analysed the area under body weight curve (Fig. 3b), as well as the Lee Index (Fig. 3c) measured at the end of the nutritional intervention period. Such decreases are, at least in part, related to the lower energy intake of HS/HP rats throughout the nutritional intervention, as compared to all the other groups (Fig. 3d, e), *p* < 0.001. On the other hand, HS/CT rats increased their energy intake back to CT/CT levels. Additionally, HPD consistently decreased white and brown adipose tissue depots in HS/HP rats, as compared to HS/HS (Table 1). Replacing HSD by standard chow also resulted in decreased adipose tissue accumulation, even though in a lesser extent than that caused by HPD (Table 1). HPD had no effect on liver weight, but increased skeletal muscle mass in comparison with the other two HSD-fed groups (Table 1).

**Table 1:** Tissues morphometry after nutritional intervention

Tissues (g/100 g BW)	CT/CT	HS/HS	HS/CT	HS/HP
Retroperitoneal fat	1.46 ± 0.13	3.09 ± 0.1^a^	2.07 ± 0.19^a,b^	1.19 ± 0.09^b,c^
Periepididymal fat	1.28 ± 0.06	3.23 ± 0.25^a^	2.19 ± 0.17^a,b^	1.58 ± 0.12^b,c^
Mesenteric fat	1.48 ± 0.16	2.88 ± 0.32^a^	2.08 ± 0.19^a,b^	1.29 ± 0.12^b,c^
Brown adipose tissue	0.12 ± 0.01	0.21 ± 0.01^a^	0.16 ± 0.01^a,b^	0.1 ± 0.01^b,c^
Liver	2.50 ± 0.06	2.55 ± 0.03	2.37 ± 0.06	2.60 ± 0.10
Skeletal muscles	1.17 ± 0.03	1.07 ± 0.02	1.09 ± 0.04	1.25 ± 0.03^b,c^

*CT/CT* rats fed a standard chow (*n* = 7), *HS/HS* rats fed a high-sucrose diet (*n* = 6), *HS/CT* rats initially fed HSD and then replaced by standard chow (*n* = 6), and *HS/HP* rats fed a HS diet and then replaced by high-protein diet (*n* = 6). Values represent mean ± SEM, compared by one-way ANOVA (Newman Keuls)

^a^represents *p* < 0.05 when compared to CT/CT

^b^represents *p* < 0.05 when compared to HS/HS

^c^represents *p* < 0.05 when compared to HS/CT

**Fig. 4:**
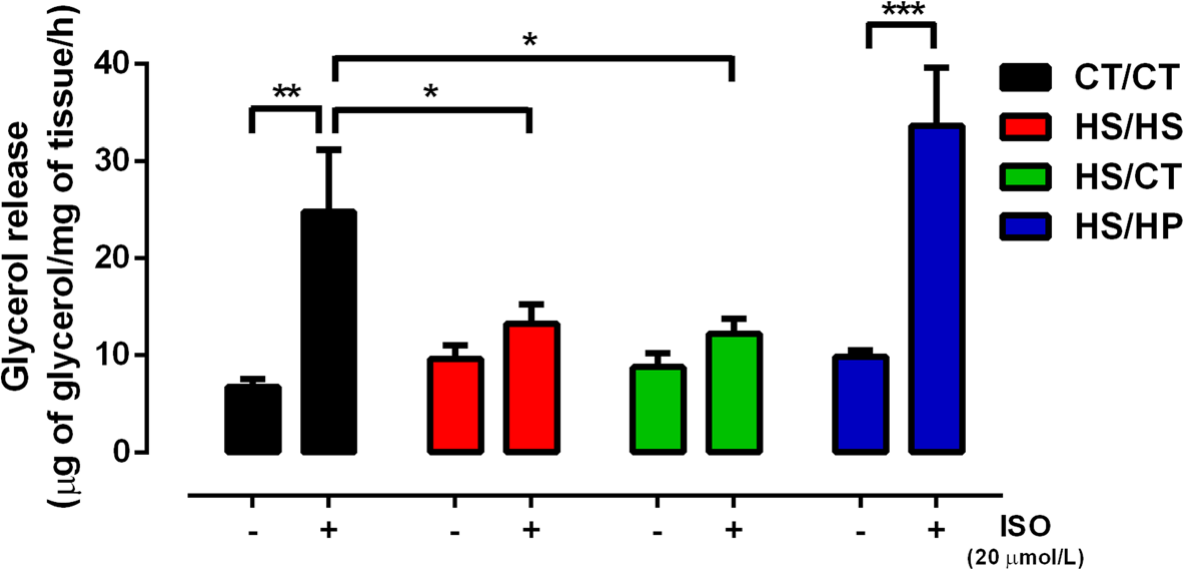
Ex vivo lipolytic activity after nutritional intervention. Glycerol release rate (μg/mg of tissue/h) from periepididymal fat pads under basal or isoproterenol (Iso; 20 μM) stimulation were assessed in rats fed a standard chow (CT/CT, *n* = 7), high-sucrose diet (HS/HS, *n* = 6), initially fed a HS diet and then replaced by standard chow (HS/CT, *n* = 6) and fed a HS diet then replaced by high-protein diet (HS/HP, *n* = 6). The nutritional intervention started after 20 weeks post weaning and lasted for 12 weeks. Bars represent mean ± SEM, compared by one-way ANOVA (Newman Keuls). ^*^ represents *p* < 0.05, ^**^ represents *p* < 0.01, ^***^ represents *p* < 0.001

To further characterize HPD effects on adiposity, we measured basal and isoproterenol-evoked ex vivo lipolysis in periepididymal fat pads from all groups. Data in Fig. 4 show that all groups had similar lipolytic activity at basal conditions, but that long-term exposure to HSD abolished the physiological lipolytic response to sympathetic stimulus. HS/HS group was incapable of rising glycerol release under isoproterenol presence, as compared to the 4-fold increase observed in CT/CT group (6.74 ± 0.82 vs. 24.81 ± 6.32 μg/mg/h, *p* < 0.01). On the other hand, HS/HP group showed a completely restored adipose tissue responsiveness to isoproterenol (9.83 ± 0.65 vs. 33.61 ± 6.01 μg/mg/h, *p* < 0.001), an effect not observed in HS/CT group, which had HSD simply replaced by standard chow (Fig. 4). This set of data emphasizes the deleterious effects of high-sucrose consumption on sympathetic-evoked lipolysis and the counter modulatory effect promoted by high-protein intake instead of the simple excess sucrose withdrawal.

### High-protein diet repairs glucose-insulin axis disruption induced by high-sucrose consumption

**Fig. 5:**
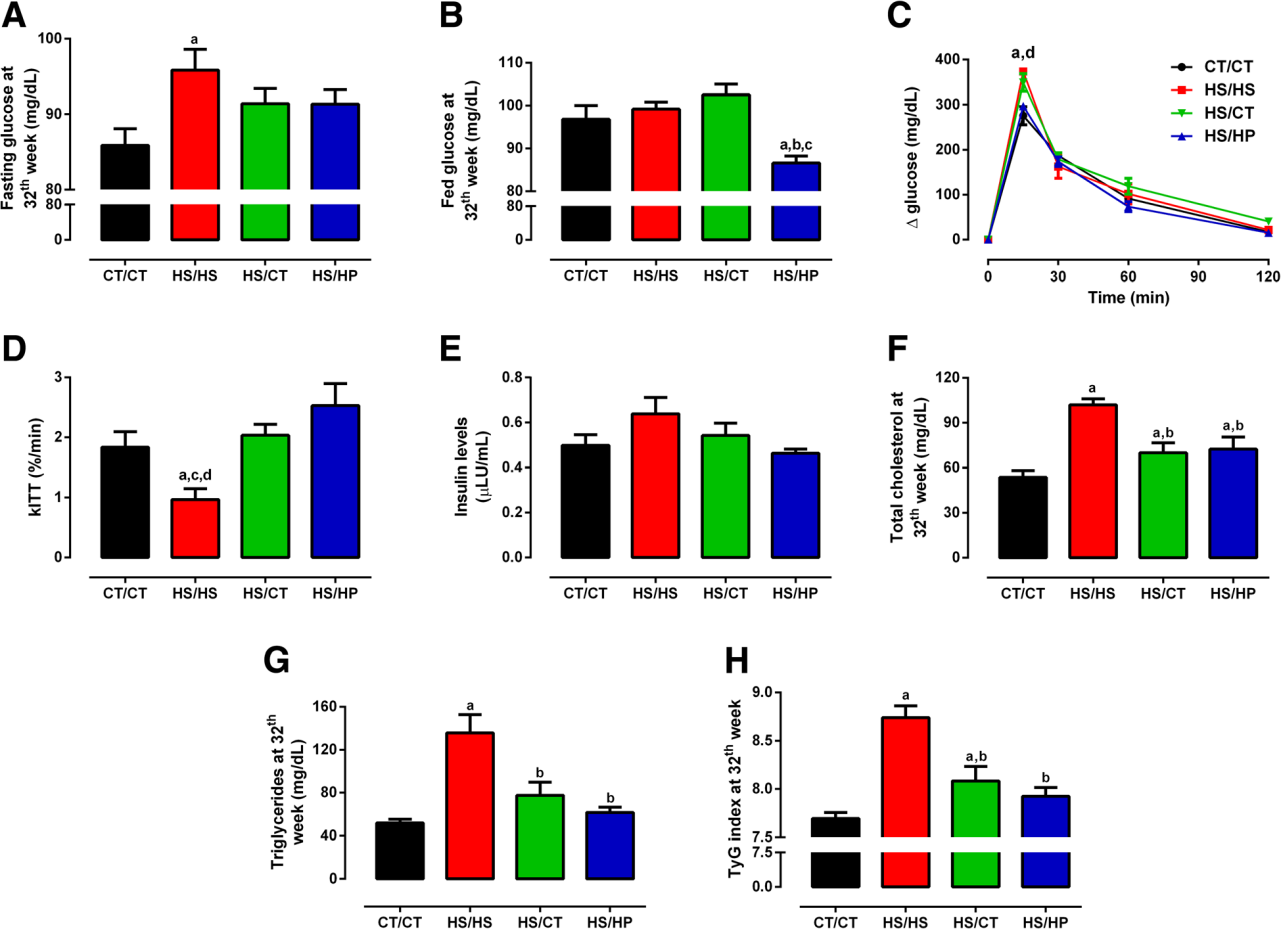
Glycemic/lipid profile and insulin resistance after nutritional intervention. **a**-**b**, blood glucose levels (mg/dL) in fasting and fed states; **c**, blood glucose levels (mg/dL) during glucose tolerance test (GTT); **d**, blood glucose disappearance rate (kITT) after insulin tolerance test (ITT); **e**, serum insulin levels (μLU/mL); **f**, serum total cholesterol levels (mg/dL); **g**, serum triglycerides levels (mg/dL); and **h**, TyG index assessed in rats fed a standard chow (CT/CT, *n* = 7), high-sucrose diet (HS/HS, *n* = 6), initially fed a HS diet and then replaced by standard chow (HS/CT, *n* = 6) and fed a HS diet then replaced by high-protein diet (HS/HP, *n* = 6). The nutritional intervention started after 20 weeks post weaning and lasted for 12 weeks. Values represent mean ± SEM, compared by one-way ANOVA (Newman Keuls). ^a^ represents *p* < 0.05 when compared to CT/CT, ^b^ represents *p* < 0.05 when compared to HS/HS, ^c^ represents *p* < 0.05 when compared to HS/CT and  ^d^ represents *p* < 0.05 when compared to HS/HP

Exposure to HPD, as well as HSD withdrawal attenuated the disruption of glucose homeostasis verified at 20th week on HSD diet. As shown in Fig. 5a, HS/HS rats presented fasting dysglycaemia in comparison to CT/CT group (95.8 ± 2.8 vs. 85.9 ± 2.2 mg/dL, *p* < 0.05), which was equally attenuated by either HPD introduction or HSD withdrawal, reaching levels not different from both HS/HS and CT/CT groups. When glucose levels were measured in fed state, only HS/HP group presented levels significantly lower than CT/CT group (86.6 ± 1.6 vs. 96.8 ± 3.2 mg/dL, *p* < 0.05; Fig. 5b). In accordance to the latter, glucose levels at 15-min peak on *ip*GTT from HS/HP group were brought back to CT/CT-like levels (*p* < 0.01), an outcome not showed by HS/CT group (Fig. 5c). Analysis of glycaemia decay during ITT (kITT) showed HS/HS rats presented impaired peripheral insulin sensitivity, which was restored by both nutritional interventions (Fig. 5d). Surprisingly, fasting serum insulin levels did not differ among groups (Fig. 5e).

Dyslipidaemia induced by long-term exposure to HSD was completely normalized upon 12-week nutritional intervention period. Fasting serum TC levels were reduced in both HS/HP and HS/CT groups (72.4 ± 8.1 and 69.9 ± 6.7 mg/dL, respectively), as compared to HS/HS group (101.9 ± 4.1 mg/dL, *p* < 0.01), which was 2-fold higher than CT/CT (53.7 ± 4.6 mg/dL, *p* < 0.001; Fig. 5f). Similarly, serum TAG levels were 2.5-fold higher in HS/HS group (135.6 ± 17.0 mg/dL, *p* < 0.001) than in CT/CT (53.0 ± 3.8 mg/dL), but were strongly reduced to 61.6 ± 4.8 mg/dL and 77.6 ± 12.2 mg/dL in HS/HP and HS/CT, respectively (*p* < 0.01; Fig. 5g). Notwithstanding, TyG Index values (Fig. 5g) indicated the maintenance of hepatic insulin resistance in HS/HS group (8.74 ± 0.06), an outcome attenuated by HSD replacement for standard chow (HS/CT, 8.08 ± 0.14; *p* < 0.001), but completely reverted in HSD-fed rats (HS/HP, 7.94 ± 0.09; *p* < 0.0001), making the latter not different from CT/CT (7.64 ± 0.06). According to these data, excess sucrose withdrawal importantly attenuates the deleterious effects of long-term sucrose exposure on glucose-insulin axis. However, its replacement by HPD promoted the same effects but further improved peripheral insulin sensitivity and glucose tolerance in the first 15 min after glucose load.

### High-protein diet regresses hepatic lipid accumulation and peroxidation

**Fig. 6:**
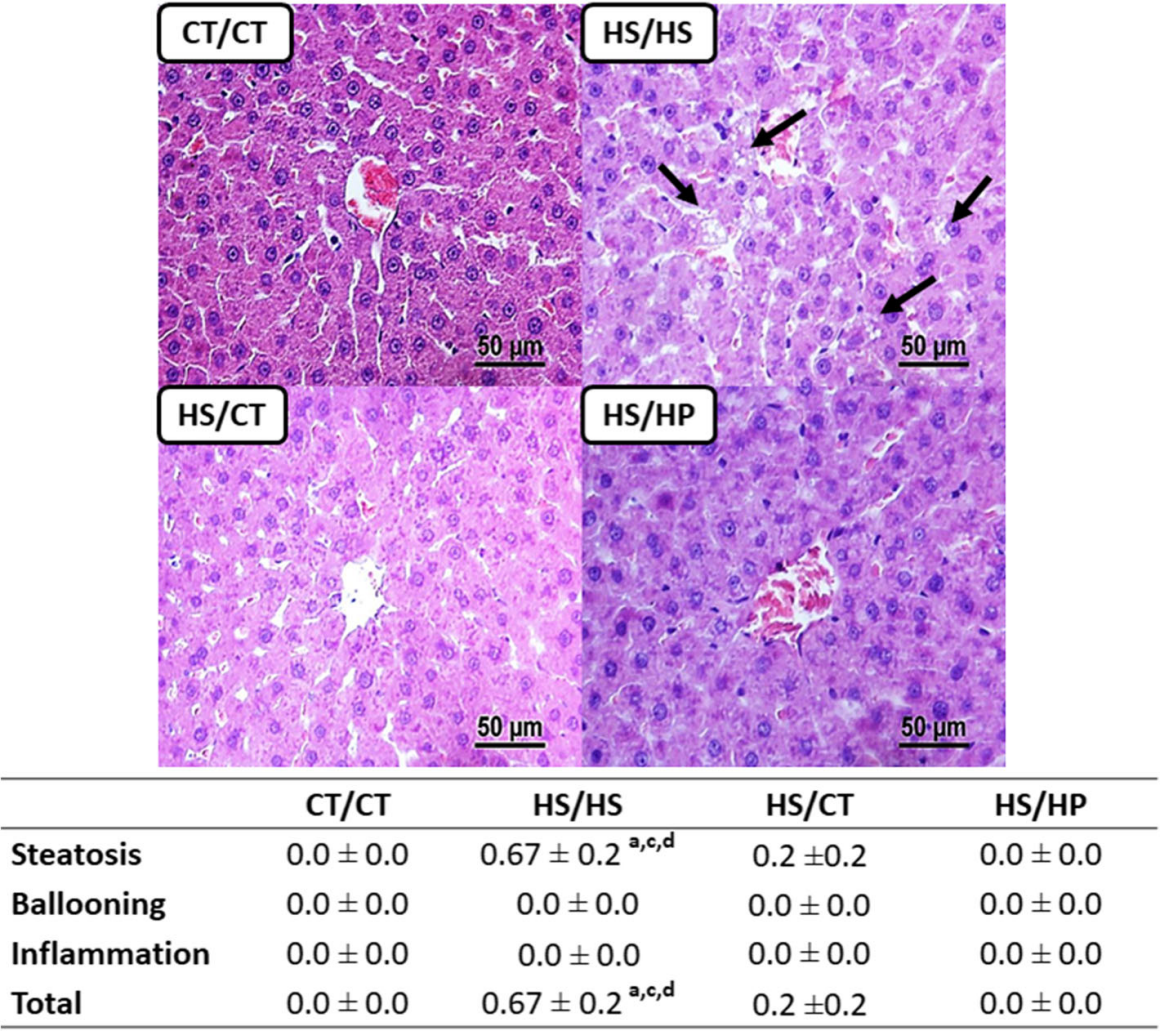
Histological analysis of livers after nutritional intervention. Sections of liver samples stained with H&E for visualization of hepatocytes morphologies and score for steatosis, ballooning and inflammation assessed in rats fed a standard chow (CT/CT, *n* = 7), high-sucrose diet (HS/HS, *n* = 6), initially fed a HS diet and then replaced by standard chow (HS/CT, *n* = 6) and fed a HS diet then replaced by high-protein diet (HS/HP, *n* = 6). The nutritional intervention started after 20 weeks post weaning and lasted for 12 weeks. Values represent mean ± SEM, compared by one-way ANOVA (Newman Keuls). ^a^ represents *p* < 0.05 when compared to CT/CT, ^c^ represents *p* < 0.05 when compared to HS/CT and ^d^ represents *p* < 0.05 when compared to HS/HP

**Fig. 7:**
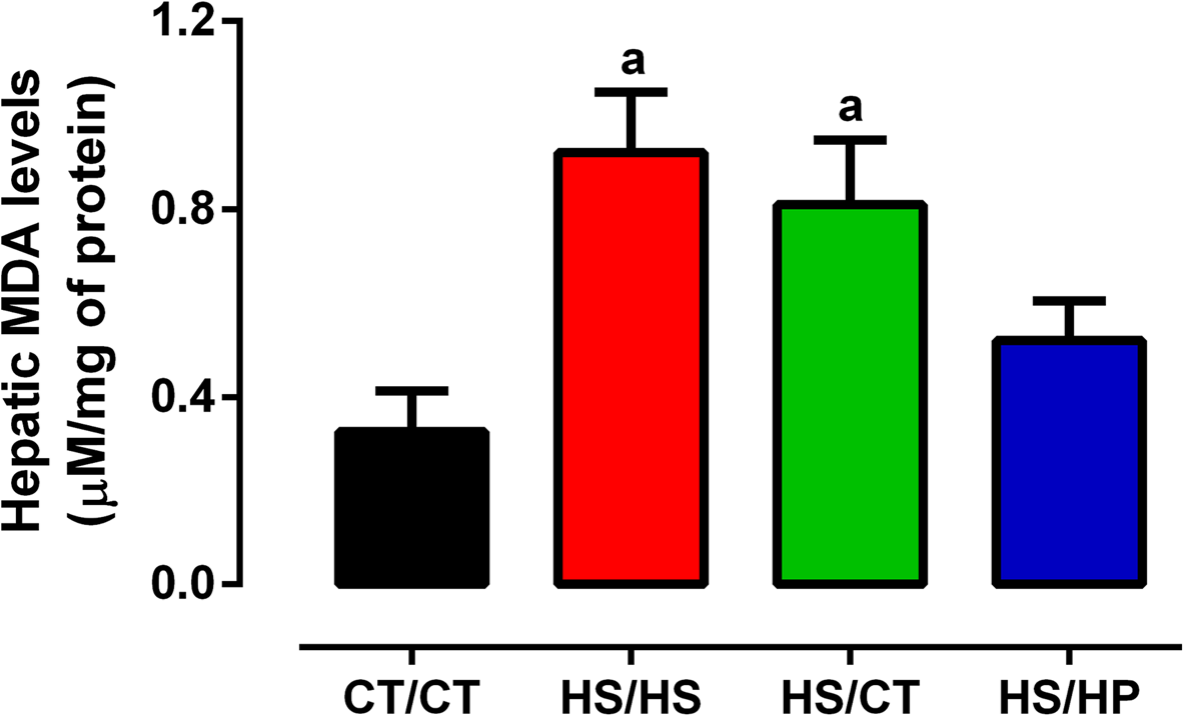
Hepatic lipoperoxidation after nutritional intervention. Spectrophotometric quantification of malondialdehyde (MDA) in homogenated samples of livers from rats fed a standard chow (CT/CT, *n* = 7), high-sucrose diet (HS/HS, *n* = 6), initially fed a HS diet and then replaced by standard chow (HS/CT, *n* = 6) and fed a HS diet then replaced by high-protein diet (HS/HP, *n* = 6). The nutritional intervention started after 20 weeks post weaning and lasted for 12 weeks. Values represent mean ± SEM, compared by one-way ANOVA (Newman Keuls). ^a^ represents *p* < 0.05 when compared to CT/CT

Microscopic analysis of H&E-stained liver slices according to the NAFLD activity score (NAS) showed that long-term exposure to HSD led to hepatic steatosis, whereas neither hepatocyte ballooning nor inflammatory infiltration were observed (Fig. 6). Because of ectopic lipid accumulation, HS/HS rats also presented increased serum ALT activity (Table 2) and 2.5 higher levels of MDA in liver (Fig. 7). Both nutritional interventions led to regression of steatosis, as well as ALT activity to CT/CT-like levels. On the other hand, only HS/HP rats presented attenuated oxidative stress, since MDA levels were not statistically different from neither HS/HS nor CT/CT rats (Fig. 7). These data reinforce the direct effects of sucrose consumption on NAFLD onset. Furthermore, they support the persistence of oxidative damage even after steatosis regression, suggesting that HPD intake seems to be more beneficial than mere high-sucrose withdrawal.

**Table 2:** Biochemical profile of hepatic function after nutritional intervention

Biochemical profile of hepatic function	CT/CT	HS/HS	HS/CT	HS/HP
AST (U/L)	27.34 ± 3.58	35.99 ± 3.66	29.04 ± 2.24	30.95 ± 4.59
ALT (U/L)	18.03 ± 0.81	30.94 ± 4.88^a,c,d^	16.83 ± 1.41	17.69 ± 1.19
γ-GT (U/L)	21.52 ± 0.52	19.62 ± 1.04	21.75 ± 0.3	22.96 ± 0.28
Alkaline phosphatase (U/L)	26.93 ± 1.77	31.48 ± 3.42	34.59 ± 2.76	32.84 ± 2.47
Albumin (g/dL)	2.43 ± 0.06	2.56 ± 0.09	2.46 ± 0.08	2.35 ± 0.09
Total protein (g/dL)	2.54 ± 0.05	2.67 ± 0.05	2.59 ± 0.02	2.68 ± 0.04
Urea (mg/dL)	48.34 ± 2.58	44.83 ± 3.45	51.42 ± 0.85	51.41 ± 2.21

*CT/CT* rats fed a standard chow (*n* = 7), *HS/HS* rats fed a high-sucrose diet (*n* = 6), *HS/CT* rats initially fed HSD and then replaced by standard chow (*n* = 6); and *HS/HP* rats fed a HS diet and then replaced by high-protein diet (*n* = 6). Values represent mean ± SEM, compared by one-way ANOVA (Newman Keuls)

^a^represents *p* < 0.05 when compared to CT/CT

^c^represents *p* < 0.05 when compared to HS/CT

^d^represents *p* < 0.05 when compared to HS/HP

## Discussion

Body weight management should be reached by simple appliance of energy balance equation, which leads to either weight loss if energy intake is less than energy expenditure, or weight gain if it occurs vice-versa. However, since meals with identical energy content may have unlimited combinations of dietary fats, carbohydrates, proteins or even alcohol; dietary manipulation has proven to be a challenging task for health professionals and billion people worldwide who are overweight or obese [13]. Most of the weight-loss diet literature has focused on the duel between low-fat and low-carbohydrate approaches, whereas high-protein interventions have currently attracted increasing attention [14, 15]. To our knowledge, the effects of long-term high-protein intake versus excess added sugar withdrawal on body weight loss and MetS-associated comorbidities have never been assessed. Consequently, we analysed two different groups of rats initially fed an HSD for 20-weeks, to then have their chow replaced by an isocaloric HPD or standard diet for additional 12-weeks to comparatively evaluate their metabolic responses.

Our results showed that post-weaning exposure to excess sucrose led HS/HS rats to progressively increase their body weight achieving an obese status, as assessed by Lee Index calculation, in accordance with previous studies from us [12] and others [33]. Intriguingly, such weight gain occurred in spite of the fact that HS/HS rats had a total energy intake significantly lower than CT/CT ones. Sucrose-derived monosaccharides, glucose and fructose, have been shown to induce malonyl-CoA expression and consequent suppression of signalling pathways activated by orexigenic neuropeptides on hypothalamic nuclei responsible for appetite control [34]. There is additional evidence that high-sucrose but not high-fat induces oxytocin-mediated mechanisms which supress certain types of feeding reward, as a kind of protection against overeating carbohydrates [35]. Despite huge literature supporting hyperphagia induced by sucrose [36–39], it has been shown that over time this hyperphagia declines and total energy intake returns to a level that resembles control [40, 41]. Thus, though we did not aim to assess feeding behaviour and reward in our current study, this set of data certainly laid the groundwork for future studies on the effect of long-term exposure to excess sucrose on appetite control.

Besides obese, HS/HS rats were dyslipidemic, glucose intolerant and insulin resistant, gathering enough risk factors to be diagnosed as suffering from MetS [42]. On the other hand, HS/HS rats only showed dysglycaemia around 28 week on HSD diet (data not shown), suggesting the Wistar-Hannover strain to be someway resistant to diet-induced hyperglycaemia. Even after 32-weeks of HSD consumption, HS/HS rats showed only mild steatosis, reinforcing the possible resistance of Wistar-Hannover rats to some of the HSD-induced metabolic outcomes. To our knowledge, this specific rat strain has not yet been assessed for its obesity proneness, although regular Wistar rats have shown greater resistance to diet-induced obesity than other rat strains, such as Wistar Kyoto and Sprague-Dawley [43–45].

Despite the abovementioned issues, the alterations herein described are primarily attributed to long-term consumption of sucrose-derived fructose. Fructose undergoes high uptake rate in an insulin-independent manner by the liver, where it is readily metabolized into TAG, not passing by the multiple control points that regulate the conversion of glucose to fat through DNL [46]. Nevertheless, glucose also plays an important role, since upregulation of hepatic DNL has been shown to be exacerbated upon glucose and fructose co-ingestion, like as in excess sucrose feeding [47]. Long-term DNL upregulation intensifies ectopic lipid accumulation that locally impairs insulin signalling [11]. Elevated serum levels of TAG and TC found in HS/HS rats are also directly associated to the impairment of hepatic insulin signalling, which closely regulates the assembly and secretion of VLDL particles [48]. The assumption of hepatic insulin resistance in HS/HS rats was further supported by the calculation of TyG Index, a surrogate method to assess insulin sensitivity [25].

Once obesity and MetS were characterized in HS/HS rats, as compared to CT/CT ones, we next tested our hypothesis. HS/HS group was split up in three different groups (HS/HS, HS/CT and HS/HP, as made explicit in Methods Section) and followed up for an additional 12-week nutritional intervention period. HPD-fed rats were the only to consistently lose weight, achieving body weight and Lee Index values slightly below those from CT/CT rats. In accordance, HS/HP rats had the lowest energy intake among all groups, which was assessed throughout the nutritional intervention. Previous studies in rodents have confirmed the capacity of diets containing up to 60% of energy as protein to reduce energy intake and body weight [17, 21, 49]. In both rats [19] and mice [17], high-protein effects on satiety have been ascribed to the action of gastrointestinal peptides, such as cholecystokinin and glucagon-like peptide 1, on the nucleus of solitary tract, while the possible role of HPD low palatability has been ruled out by different studies [17, 19, 50, 51].

Noteworthy, our HPD-fed rats did not regain body weight, as did in other studies with identical nutritional intervention period [17, 20, 52, 53]. This finding may be related to the fact that our HPD contained nearly 35% of energy as protein, whereas those used in the aforementioned studies had a minimum of 48%. To our knowledge, no study has compared long-term effects of diets containing different high-protein levels on satiety and body weight management. Nevertheless, we speculate that long-term very high intake of dietary proteins would activate feedback mechanisms to restore carbohydrate-driven rewarding feeding behaviour, in an opposite way to that proposed for high-sucrose intake [35]. On the other hand, HS/CT rats did not reduce body weight nor energy intake, but rather kept an upward trend of weight gain in parallel with HS/HS ones. Although contradictory, it has been shown that sometimes high-fat diet-induced body weight gain is defended completely when dietary palatability is reduced, for instance by giving standard chow back, whereas others give rise to additional body weight that is only partially defended [54]. There is poor literature on the mechanisms involved in body weight defence upon withdrawal from long-term high-sucrose consumption, however the involvement of hedonic systems has been proposed as the most likely candidate for such effect [54, 55].

Long-term exposure to HPD, as well as withdrawal from HSD promoted very similar metabolic outcomes upon 12-week nutritional intervention. HS/HP and HS/CT rats showed reduced fasting serum levels of glucose, TAG and TC, which are directly correlated with the improvement of peripheral insulin sensitivity, as inferred from *k*ITT and TyG Index values. Accordingly, lowering sucrose intake also resulted in return of hepatic steatosis and serum ALT activity back to control patterns. These outcomes are mainly ascribed to the counter-modulatory effect of the lower sucrose-derived fructose content in the standard chow, which is indeed absent in HPD, allowing downregulation of DNL-related genes, reestablishment of hepatic insulin sensitivity and reduced assembly and secretion of VLDL particles [46, 48]. Besides, long-term HPD intake per se has been shown also to downregulate lipogenic genes expression in both liver and white adipose tissue from HSD fed rats [56]. On the other hand, only HPD-fed rats presented significantly lower serum glucose levels at 15-min *ip*GTT peak and fed state as well. This augmented glucose handling capacity might be directly associated to the increased skeletal muscle mass found only in HS/HP group, as previously demonstrated elsewhere [16, 56]. Surprisingly, hepatic lipid peroxidation was attenuated only in HS/HP rats, supporting previous report on the protective action of long-term HPD intake against oxidative stress damage in liver [57].

Last but not least, long-term HPD intake completely reverted WAT accumulation, whereas it was only partially reduced upon withdrawal from HSD, in agreement with previously reported studies [51, 56]. These results can be explained to some extent by the reduction in caloric intake on HS/HP rats. However, the subsequent huge regression of all measured fat pads could also arise from an increased thermogenic response to a high-protein intake. The long-term effect of HPDs on total energy expenditure and metabolic rate is still a matter of intense debate [17, 52, 53, 56]. However, there is consistent evidence for the stimulating role of dietary high-protein consumption on the WAT responsiveness to the lipolytic action of sympathetic agonists [58], which is importantly impaired in diet-induced obese rats [59, 60]. Moreover, it has been suggested that vagus nerve afferences carry information relative to the quantity of protein ingested to hypothalamic sites responsible for food intake control, which may alter peripheral sympathetic nervous system activity [61]. Thus, given the complete rescue of WAT sensitivity to isoproterenol observed in HS/HP, but not HS/CT rats, these data consistently support high-protein intake as an important strategy for adiposity decrease and weight loss in the context of MetS.

## Conclusions

Although we have prioritized functional instead of molecular approaches in this study, our data importantly show that 12-week intake of an isocaloric moderately high-protein diet consistently restored high-sucrose-induced central adiposity and obesity in addition to the attenuation of other important metabolic outcomes, such as improvement of glucolipid homeostasis associated to increased insulin sensitivity and reversal of hepatic steatosis. On the other hand, simple withdrawal from high-sucrose consumption also promoted the abovementioned metabolic outcomes with no impact on body weight. Thus, both nutritional interventions show themselves to be mutually efficient, potentially helping health professionals to make better decisions on how to treat patients with MetS associated or not to overweight/obesity.

## Additional file

Additional file 1: **Table S1.** Micronutrient composition of diets. (PDF 79 kb)

## Abbreviations

CT: Control diet
DNL: De novo lipogenesis
HP: High-protein diet
HS: High-sucrose diet
*ip*GTT: Intraperitoneal glucose tolerance test
*ip*ITT: Intraperitoneal insulin tolerance test
MetS: Metabolic syndrome
NAFLD: Non-alcoholic fatty liver disease
NAS: NAFLD activity score
T2DM: Type 2 diabetes mellitus
TAG: Triacylglicerol
TC: Total cholesterol
WAT: White adipose tissue

## References

[1] Bray GA, Popkin BM (2014). Dietary sugar and body weight: have we reached a crisis in the epidemic of obesity and diabetes?: health be damned! Pour on the sugar. Diabetes Care.

[2] Kahn R, Sievenpiper JL (2014). Dietary sugar and body weight: have we reached a crisis in the epidemic of obesity and diabetes?: we have, but the pox on sugar is overwrought and overworked. Diabetes Care.

[3] Stanhope KL (2016). Sugar consumption, metabolic disease and obesity: the state of the controversy. Crit Rev Clin Lab Sci.

[4] Aydin S, Aksoy A, Aydin S, Kalayci M, Yilmaz M, Kuloglu T, Citil C, Catak Z (2014). Today's and yesterday's of pathophysiology: biochemistry of metabolic syndrome and animal models. Nutrition.

[5] Narain A, Kwok CS, Mamas MA. Soft drink intake and the risk of metabolic syndrome: a systematic review and meta-analysis. Int J Clin Pract. 2017;71 10.1111/ijcp.12927 28074617

[6] Johnson RJ, Lanaspa MA, Sanchez-Lozada LG, Rivard CJ, Bjornstad PS, Merriman T, Sundborn G (2014). Fat storage syndrome in Pacific peoples: a combination of environment and genetics?. Pac Health Dialog.

[7] Te Morenga L, Mallard S, Mann J (2012). Dietary sugars and body weight: systematic review and meta-analyses of randomised controlled trials and cohort studies. BMJ.

[8] Tappy L, Le KA, Tran C, Paquot N (2010). Fructose and metabolic diseases: new findings, new questions. Nutrition.

[9] Parks EJ, Skokan LE, Timlin MT, Dingfelder CS (2008). Dietary sugars stimulate fatty acid synthesis in adults. J Nutr.

[10] Masoodi M, Kuda O, Rossmeisl M, Flachs P, Kopecky J (2015). Lipid signaling in adipose tissue: connecting inflammation & metabolism. Biochim Biophys Acta.

[11] Chen Z, Yu R, Xiong Y, Du F, Zhu S (2017). A vicious circle between insulin resistance and inflammation in nonalcoholic fatty liver disease. Lipids Health Dis.

[12] Pinto BAS, Melo TM, Flister KFT, França LM, Kajihara D, Tanaka LY, Laurindo FRM, de Andrade Paes AM. Early and sustained exposure to high-sucrose diet triggers hippocampal ER stress in young rats. Metab Brain Dis. 2016:1–11. 10.1007/s11011-016-9830-1 27154727

[13] Gardner CD (2012). Tailoring dietary approaches for weight loss. Int J Obes Suppl.

[14] Campos-Nonato I, Hernandez L, Barquera S (2017). Effect of a high-protein diet versus standard-protein diet on weight loss and biomarkers of metabolic syndrome: a randomized clinical trial. Obes Facts.

[15] Bray GA, Ryan DH, Johnson W, Champagne CM, Johnson CM, Rood J, Williamson DA, Sacks FM (2017). Markers of dietary protein intake are associated with successful weight loss in the POUNDS lost trial. Clin Obes.

[16] French WW, Dridi S, Shouse SA, Wu H, Hawley A, Lee SO, Gu X, Baum JI. A high-protein diet reduces weight gain, decreases food intake, decreases liver fat deposition, and improves markers of muscle metabolism in obese Zucker rats. Nutrients. 2017;9 10.3390/nu9060587 PMC5490566 28594375

[17] Vu JP, Luong L, Parsons WF, Oh S, Sanford D, Gabalski A, Lighton JR, Pisegna JR, Germano PM (2017). Long-term intake of a high-protein diet affects body phenotype, metabolism, and plasma hormones in mice. J Nutr.

[18] Potier M, Darcel N, Tome D (2009). Protein, amino acids and the control of food intake. Curr Opin Clin Nutr Metab Care.

[19] Faipoux R, Tome D, Gougis S, Darcel N, Fromentin G (2008). Proteins activate satiety-related neuronal pathways in the brainstem and hypothalamus of rats. J Nutr.

[20] Clifton P (2012). Effects of a high protein diet on body weight and comorbidities associated with obesity. Br J Nutr.

[21] Jean C, Rome S, Mathe V, Huneau JF, Aattouri N, Fromentin G, Achagiotis CL, Tome D (2001). Metabolic evidence for adaptation to a high protein diet in rats. J Nutr.

[22] Greenberg I, Stampfer MJ, Schwarzfuchs D, Shai I, Group D (2009). Adherence and success in long-term weight loss diets: the dietary intervention randomized controlled trial (DIRECT). J Am Coll Nutr.

[23] Bernardis LL, Patterson BD (1968). Correlation between 'Lee index' and carcass fat content in weanling and adult female rats with hypothalamic lesions. J Endocrinol.

[24] Rafacho A, Abrantes JL, Ribeiro DL, Paula FM, Pinto ME, Boschero AC, Bosqueiro JR (2011). Morphofunctional alterations in endocrine pancreas of short- and long-term dexamethasone-treated rats. Horm Metab Res.

[25] Simental-Mendia LE, Rodriguez-Moran M, Guerrero-Romero F (2008). The product of fasting glucose and triglycerides as surrogate for identifying insulin resistance in apparently healthy subjects. Metab Syndr Relat Disord.

[26] Vaughan M (1962). The production and release of glycerol by adipose tissue incubated in vitro. J Biol Chem.

[27] Mihara M, Uchiyama M (1978). Determination of malonaldehyde precursor in tissues by thiobarbituric acid test. Anal Biochem.

[28] Moore K, Roberts LJ (1998). 2nd: Measurement of lipid peroxidation. Free Radic Res.

[29] Kleiner DE, Brunt EM, Van Natta M, Behling C, Contos MJ, Cummings OW, Ferrell LD, Liu YC, Torbenson MS, Unalp-Arida A (2005). Design and validation of a histological scoring system for nonalcoholic fatty liver disease. Hepatology.

[30] Faul F, Erdfelder E, Lang AG, Buchner A (2007). G*power 3: a flexible statistical power analysis program for the social, behavioral, and biomedical sciences. Behav Res Methods.

[31] Uebanso T, Taketani Y, Fukaya M, Sato K, Takei Y, Sato T, Sawada N, Amo K, Harada N, Arai H (2009). Hypocaloric high-protein diet improves fatty liver and hypertriglyceridemia in sucrose-fed obese rats via two pathways. Am J Physiol Endocrinol Metab.

[32] Diaz-Rua R, Keijer J, Palou A, van Schothorst EM, Oliver P (2017). Long-term intake of a high-protein diet increases liver triacylglycerol deposition pathways and hepatic signs of injury in rats. J Nutr Biochem.

[33] de Queiroz KB, Coimbra RS, Ferreira AR, Carneiro CM, Paiva NC, Costa DC, Evangelista EA, Guerra-Sa R (2014). Molecular mechanism driving retroperitoneal adipocyte hypertrophy and hyperplasia in response to a high-sugar diet. Mol Nutr Food Res.

[34] Lane MD, Cha SH (2009). Effect of glucose and fructose on food intake via malonyl-CoA signaling in the brain. Biochem Biophys Res Commun.

[35] Klockars A, Levine AS, Olszewski PK (2015). Central oxytocin and food intake: focus on macronutrient-driven reward. Front Endocrinol (Lausanne).

[36] Lindqvist A, de la Cour CD, Stegmark A, Hakanson R, Erlanson-Albertsson C (2005). Overeating of palatable food is associated with blunted leptin and ghrelin responses. Regul Pept.

[37] Inam QU, Jabeen B, Haleem MA, Haleem DJ (2008). Long-term consumption of sugar-rich diet decreases the effectiveness of somatodendritic serotonin-1A receptors. Nutr Neurosci.

[38] Gaysinskaya VA, Karatayev O, Shuluk J, Leibowitz SF (2011). Hyperphagia induced by sucrose: relation to circulating and CSF glucose and corticosterone and orexigenic peptides in the arcuate nucleus. Pharmacol Biochem Behav.

[39] Treesukosol Y, Liang NC, Moran TH (2015). Alterations in sucrose sham-feeding intake as a function of diet-exposure in rats maintained on calorically dense diets. Appetite.

[40] Sclafani A, Lucas F, Ackroff K (1996). The importance of taste and palatability in carbohydrate-induced overeating in rats. Am J Phys.

[41] Colley DL, Castonguay TW (2015). Effects of sugar solutions on hypothalamic appetite regulation. Physiol Behav.

[42] Alberti KG, Zimmet P, Shaw J, Group IDFETFC (2005). The metabolic syndrome--a new worldwide definition. Lancet.

[43] Akieda-Asai S, Koda S, Sugiyama M, Hasegawa K, Furuya M, Miyazato M, Date Y (2013). Metabolic features of rats resistant to a high-fat diet. Obes Res Clin Pract.

[44] de Artinano AA, Castro MM (2009). Experimental rat models to study the metabolic syndrome. Br J Nutr.

[45] Azzout-Marniche D, Chaumontet C, Nadkarni NA, Piedcoq J, Fromentin G, Tome D, Even PC (2014). Food intake and energy expenditure are increased in high-fat-sensitive but not in high-carbohydrate-sensitive obesity-prone rats. Am J Physiol Regul Integr Comp Physiol.

[46] Tappy L, Le KA (2010). Metabolic effects of fructose and the worldwide increase in obesity. Physiol Rev.

[47] Kolderup A, Svihus B (2015). Fructose metabolism and relation to atherosclerosis, type 2 diabetes, and obesity. J Nutr Metab.

[48] Kamagate A, Dong HH (2008). FoxO1 integrates insulin signaling to VLDL production. Cell Cycle.

[49] Stengel A, Goebel-Stengel M, Wang L, Hu E, Karasawa H, Pisegna JR, Tache Y (2013). High-protein diet selectively reduces fat mass and improves glucose tolerance in western-type diet-induced obese rats. Am J Physiol Regul Integr Comp Physiol.

[50] L'Heureux-Bouron D, Tome D, Bensaid A, Morens C, Gaudichon C, Fromentin G (2004). A very high 70%-protein diet does not induce conditioned taste aversion in rats. J Nutr.

[51] Lacroix M, Gaudichon C, Martin A, Morens C, Mathe V, Tome D, Huneau JF (2004). A long-term high-protein diet markedly reduces adipose tissue without major side effects in Wistar male rats. Am J Physiol Regul Integr Comp Physiol.

[52] Kim JH, Park Y, Kim D, Park Y (2012). Dietary influences on nonexercise physical activity and energy expenditure in C57BL/6J mice. J Food Sci.

[53] Schwarz J, Tome D, Baars A, Hooiveld GJ, Muller M (2012). Dietary protein affects gene expression and prevents lipid accumulation in the liver in mice. PLoS One.

[54] Mercer JG, Duncan JS, Archer ZA (2014). Hypothalamic gene expression during voluntary hypophagia in the Sprague-Dawley rat on withdrawal of the palatable liquid diet, ensure. Physiol Behav.

[55] Cottone P, Sabino V, Steardo L, Zorrilla EP (2008). Opioid-dependent anticipatory negative contrast and binge-like eating in rats with limited access to highly preferred food. Neuropsychopharmacology.

[56] Chaumontet C, Even PC, Schwarz J, Simonin-Foucault A, Piedcoq J, Fromentin G, Azzout-Marniche D, Tome D (2015). High dietary protein decreases fat deposition induced by high-fat and high-sucrose diet in rats. Br J Nutr.

[57] Petzke KJ, Elsner A, Proll J, Thielecke F, Metges CC (2000). Long-term high protein intake does not increase oxidative stress in rats. J Nutr.

[58] Kettelhut IC, Foss MC, Migliorini RH (1985). Lipolysis and the antilipolytic effect of insulin in adipocytes from rats adapted to a high-protein diet. Metabolism.

[59] Portillo MP, Simon E, Garcia-Calonge MA, Del Barrio AS (1999). Effect of high-fat diet on lypolisis in isolated adipocytes from visceral and subcutaneous WAT. Eur J Nutr.

[60] Jocken JW, Blaak EE (2008). Catecholamine-induced lipolysis in adipose tissue and skeletal muscle in obesity. Physiol Behav.

[61] Darcel N, Fromentin G, Raybould HE, Gougis S, Gietzen DW, Tome D (2005). Fos-positive neurons are increased in the nucleus of the solitary tract and decreased in the ventromedial hypothalamus and amygdala by a high-protein diet in rats. J Nutr.

