# Supplementary material for: Long-term high-protein diet intake reverts weight gain and attenuates metabolic dysfunction on high-sucrose-fed adult rats
Source: Nutr Metab (Lond). 2018 Jul 24;15:53. doi: 10.1186/s12986-018-0290-y (PMC6057058; doi:10.1186/s12986-018-0290-y)
Supplement: Supplementary file 1 — Table S1. Micronutrient composition of diets. (PDF 79 kb) [file 12986_2018_290_MOESM1_ESM.pdf]

Supplementary Table 1: Micronutrient composition of diets.

Micronutrients	CTR	HSD	HPD
Minerals			
Calcium (mg)	1000	400	920
Chlorine (mg)	400	160	-
Cobalt (mg)	0.15	0.06	-
Copper (mg)	1	0.4	-
Fluorine (mg)	8	3.2	-
Iodine (mg)	0.2	0.08	-
Iron (mg)	5	2	23.54
Magnesium (mg)	37	14	19.32
Manganese (mg)	6	2.4	0.55
Phosphorus(mg)	80	32	92
Potassium (mg)	1060	420	1100
Selenium(mg)	0.005	0.002	-
Sodium (mg)	270	152	92
Zinc (mg)	6	2.4	2.02
Vitamins			
Biotin (mg)	0.005	0.002	-
Choline (mg)	190	76	60
Folic acid (mg)	0.1	0.04	0.2
Niacin (mg)	6	2.4	3.0
Riboflavin (mg)	-	-	0.6
Vitamin A (IU)	1300	520	400
Vitamin B1 (mg)	0.5	0.2	0.6
Vitamin B12 (μg)	2.2	0.88	2.5
Vitamin B2 (mg)	0.6	0.24	-
Vitamin B6 (mg)	0.7	0.28	0.7
Vitamin D3 (IU)	200	80	100
Vitamin E (IU)	3.4	1.36	7.5
Vitamin K3 (mg)	0.3	0.12	0.0001

CTR, standard chow; HSD, high-sucrose diet; HPD, high-protein diet.
